# Surgical Club of S.W. England

**Published:** 1991-06

**Authors:** 


### Surgical Club of the South West

### Autumn Meeting at Weston-super-Mare, October 19th 1990

Abstracts of papers delivered

### ANGELCHICK REVISITED

A.L. Gough
Weston-s-Mare General Hospital

Dr. Angelchick published the first report of an entirely new concept in the surgical treatment of oesophageal reflux in 1979. This method involves placing a small silicone collar around the lower oesophagus below the diaphragm.

During the years 1983–1987, 36 patients who were complaining of uncontrollable oesophageal reflux were treated by this method at Weston-super-Mare General Hospital. Most of them had had symptoms for at least five years. All had oesophagogastroscopy prior to surgery and all had a hiatus hernia. 15 also had oesophagitis, 2 had Barrett's oesophagus, and 6 had strictures. All the patients tolerated the procedure well and were discharged home to be followed up in the usual way.

For the purpose of this study, a questionnaire follow up which was sent to each of them, asking whether they had — Heartburn, Reflux, or, Difficulty with swallowing. 30 patients replied, in addition to this — 3 had died and 3 could not be traced. 77% of the patients who replied reported no, or very occasional symptoms. 23% had regular symptoms. The pateints who were deemed to have a less than satisfactory result were evaluated more fully. In 2 the prosthesis had migrated through the diaphragm and into the chest. 3 had unacceptable dysphagia associated with the weight of the prosthesis. 1 — a Down's syndrome boy had episodes of vomiting and another had had a previous vagotomy and antrectomy and was troubled by bile reflux.

Our conclusions were that the Angelchick anti-reflux prosthesis is safe and gives satisfacotry results.

### ULTRASOUND VS. I.V.U.; WHICH WHEN?

P.G.P. Stoddart
Weston-super-Mare General Hospital

The choice of initial radiological investigation of the urinary tract has been changed over the last 5 years by the introduction of good quality ultrasound services. Where such a service is available, consultant based, and adequately resourced, it has replaced the I.V.U. for some urological problems. In these cases it offers a cheap, quick, painless, and safe examination without any loss of diagnostic quality. A KUB to complement the ultrasound may be advisable if there is a possibility of urinary stones. Small kidney stones, and most ureteric stones, are not reliably visible on ultrasound.

There are some conditions where the investigations are complimentary, and the initial choice will depend on local expertise and agreement. Haematuria is a good example of this; ultrasound will detect the majority of causes requiring surgical treatment, but a patient with persisting haematuria may need both cystoscopy and an IVU. The assessment of obstruction is another case where both techniques have a role. An ultrasound will detect most cases of upper tract dilatation, but can not assess the degree of hydrostatic obstruction; an IVU can show the level of obstruction, and if it is partial or absolute.

EXAMINATION OF CHOICE FOR INITIAL EXAMINATION OF URINARY TRACT

ULTRASOUND +/– KUB

Haematuria
Prostatic symptoms
Infection
Renal and bladder masses
Renal Failure
Cortical scarring

I.V.U.

Renal Colic
Stone related disease
Ureteric Disease
Assessment of severity of obstruction
Pelvi-calyceal disease

### LAPAROSCOPIC CHOLECYSTECTOMY — A NEW OPERATION

**H.J. Espiner, W.K. Eltringham, R. Miller and A.M. Rowe**
Bristol Royal Infirmary

With the development of chip cameras, good light sources and high-flow insufflators, laparoscopic abdominal surgery beyond the pelvis has become a reality. From the end of June until mid-October 1990, we carried out this procedure in 22 cases and were able to complete the operation in each instance. A standard laparoscopy was carried out via the umbilicus after catheterisation of the bladder and passage of a naso-gastric tube. Operating ports were then placed under direct vision in the upper abdomen. The first on the costal margin in the mid-clavicular line to hold the fundus of the gallbladder temporarily while a second port was placed in the anterior axilliary line above the anterior superior spine or level with the umbilicus to take the fundus and rotate the liver upwards showing the gallbladder on its inferior surface. A third and larger port for operating instruments was placed in the upper midline again under direct vision with the cannula entering the abdomen to the right of the falciform ligament. With the mid-clavicular forceps holding the neck of the gallbladder tense anteriorly inferiorly and to the right it was possible to dissect the peritoneal and vascular attachments from the gallbladder wall using the upper midline port. We adapted our fundus first technique for the laparoscopic approach and this meant separating the peritoneum and vascular connections well on the gallbladder wall over the lower third of the gallbladder initially, then across Hartmann's pouch to the neck and thence down the outer aspect of the duct. Diathermy forceps were used to seal all the small vessels under direct vision and the tissues were freed progressively until a window was created around the whole of the lower third of the gallbladder and the upper cystic duct. In this way no attempt was made to clip or divide the cystic artery and no confusion with the anatomy was possible. Three clips were then placed around the cystic duct near the neck and a laser probe was used to divide the duct between the outer two. Dissection of the gallbladder in a retrograde way from the neck to the fundus was completed by a combination of laser and diathermy hemostasis and dissection, and a special retrieval pouch was designed and used to remove the gallbladder safely contained through the upper midline port. It was possible to complete this procedure in cases of resolving cholecystitis and even mucocele and later some with empyema. Some of the more inflamed gallbladders were dealt with successfully by piecemeal removal and the contents of dissection placed in the retrieval pouch as the operation progressed.

Most of the patients were able to go home the day following surgery, although some of the older ones remained a day or two longer. One patient required re-admission with abdominal pain and at first a bile leak was suspected but not confirmed on computerised scanning.

Subsequent left lower lobar collapse was diagnosed and this responded to antibiotics. There were no other significant complications.

It was our distinct impression that the procedure represents a significant advance in biliary surgery and will surely contribute to the development of similar techniques in abdominal surgery as a whole.

### ARABIAN SANDS

### Four Years Experience in Saudi Arabia

**D.R.W. Hartley**
Weston-super-Mare General Hospital

Between 1978 and 1982 I spent four years in Saudi Arabia in 3 hospitals along its west coast — at Tabuk in the north, Khamis Mushayt in the south and the city of Jeddah in the centre.

Saudi Arabia is a unique country in several ways: it is the only one named after a family (the Saud family); it is huge in area (almost the size of Europe) but has a small population of only 8 million, all Muslim. Most of the country is desert, the people living largely around the edge, by the sea. The Saudis are the guardians of the shrine of Islam, the Holy Stone at Mecca. There is no tourism in Saudi Arabia — the only foreigners are ex-patriate employees and their families, or pilgrims visiting Mecca. Non-muslims cannot own property there; they can own businesses, but only jointly with muslim directors who must have a majority shareholding. In my view, only the Saudis and others living in the Arabian peninsula are the true Arabs — other Middle East countries loosely described as "Arabic" are Arabic-speaking only, but are themselves quick to point out their individuality. The British have always enjoyed good relations with the Saudis, historically. We have never been to war against them, and have allied with them at least twice in major conflicts.

Saudi Arabia has had to cope with the transition from rural desert poverty to colossal wealth and technology following oil discovery, within a generation and has managed so far rather better than most other Levantine and Arabic-speaking nations having the same experience. Needless to say, these changes have had profound permanent sociological effects.

There is little general practitioner service in Saudi Arabia, either urban or rural, such as we take for granted here. The sick Saudi has to get himself or herself or her child to a hospital; the wealthy to a private hospital, ordinary folk to a government hospital; and other hospitals cater especially for government employees and their families, such as the military, the police and civil service.

We anslysed 1,000 consecutive obstetric cases delivered at Khamis Mushayt in 1978[2]. A "booked" patient was defined as one we had seen once or more ante-natally. Half the patients were seen for the first time when in labour. 760 were multipara, and of these 25% had more than 6 children — the most being 13. If the 240 primipara 30% were 17 years old or less, the youngest being 12. The forceps and ventouse rate was 3.8%, and the Caesarian section rate 8.0%, comparable to the United Kingdom rates. The perinatal death rate was 17 per 1000.

Induction rates were very low, and were mostly performed for pre-eclampsia or hypertension. There were no inductions performed for post-maturity, as this was generally impossible to diagnose with the paucity of ante-natal visits and lack of patients' memories regarding timing of menses. It did not seem to make much difference to the perinatal mortality that our induction rate was 3% at a time when some units in the United Kingdom had rates of 50%.

The gynaecology spectrum was rather different from that seen in the West. Women married in their teens to men rather older than themselves, who had required the time to save money for the purchase of a wife. A wife is her husband's property, therefore, and her chief function is to produce children. Failure to do so therefore means that she has failed in her allotted task in life. Thus there is an obsession with fertility, and the commonest outpatient request is for full infertility investigations in a young, recently married women, presenting with "generalised pains". There is strong objection to hysterectomy for any reason whatsoever, for the obvious reason that an infertile woman will lose her status and probably her husband and hence her security. We did not see much pelvic sepsis, and only a few ectopic pregnancies, but spontaneous abortions are common. Naturally requests for sterilisations are few and can only be performed for medical reasons, and with the agreement of a muslim doctor. Laws governing terminations of pregnancy are brutally simple: it is legal if performed to save the patient's life — for any other reason it is illegal and punishable by death for both patient and the person performing the operation. And yet Saudies could buy a selection of oral contraceptives without a doctor's prescription in any chemist.

The medical milieu is different — the area is subtropical, so one needed to remember that bilharzia and malaria were endemic, the population is 20% positive to hepatitis B, and tuberculosis is present.

There is no litigation in Saudi medical practice — how can there be, if what befalls is the will of Allah?

The Arabian peninsula is a different experience and has much to offer those who venture there, unprejudiced and free of pre-conceived ideas. The people have a long history, a culture and values strange to us, superb rugged scenery, coral reefs teeming with life, all under a clear sky in which the milky way glows like a strip of nocturnal neon.

*References*
1. Sir J. B. Glubb, "Britain and the Arabs," Hodder & Stoughton.
2. One thousand obstetric deliveries in the Asir Province, Kingdom of Saudi Arabia. *Saudi Medical Journal*, Vol 1, No 4, pp. 187–196.

### "BORING OLD PROSTATES"

**A. Hinchcliffe**
Weston-super-Mare General Hospital

A personal series of 132 videos and 2 retropubic prostatectomies in 1989 was analysed after a 6–18 month follow up period. 21% of patients were aged over 80 and 47% of operations were for acute or chronic retention. Peri-operative mortality was nil but 12 of 14 deaths in the follow up period were in those with acute retention. (7 had advanced prostatic carcinoma, 5 cardiovascular or pulmonary causes).

Suprapubic catheters for relief of acute retention were preferred for ease of voiding trials but only 5 of 25 trials succeeded in the medium term.

Waiting list patients were all selected after flowmetry, ultrasound residual and frequency volume charts but when those with complications such as haematuria, prostatic carcinoma, raised PSA, stone, etc., had been given priority only 30 patients with significant "uncomplicated prostatism" were treated.

*Conclusions:*

1. Video prostatectomy has many advantages in tuition and for the operator.
2. Acute retainers spent many days in hospital, often for trials of voiding which were unsuccessful.
3. Carcinoma of the prostate is still presenting late but prostate specific antigen is beginning to discover more patients suitable for radical treatment.
4. Those with uncomplicated prostatism have difficulty in obtaining surgical treatment.

### FROM OBSCURITY TO EMINENCE — THE HISTORY OF THE MEDICAL SERVICES IN WESTON-SUPER-MARE, WOODSPRING

**P.H. Roberts**
Weston-super-Mare General Hospital

Dispensaries, funded by voluntary subscriptions, were started in the 18th Century, the first one in Bristol being founded in 1775. In January 1854 subscriptions were invited for a free dispensary for the population of Weston. By the 17th June, 1854 it is recorded that 442 patients had been treated in this dispensary. However dispensaries provided only out patient treatment and could not cater for serious injuries, these patients having to go to Bristol where the Infirmary had been founded in 1737 and the General Hospital in 1831.

On Monday 5 October, 1863 a public tea and soiree took place in the town hall with the purpose of enabling all classes to contribute towards a Casualty Ward Fund "and right heartily it was responded to''.

A site for the hospital was found and the new building, with 7 beds, designed by the well known local architect Hans Price, was opened in 1867.

The hospital enlarged over the years and by the first world war when military cases were admitted there were 41 beds. This hospital, known as the General Hospital, was considerably enlarged in 1928 when £60,000 was raised by public subscription this being inspired by a local businessman, Mr Henry Butt. The hospital remained in use until the opening of the new hospital in 1986.

A further hospital, initially called the West of England Sanatorium was opened on the sea front in 1870 and this was enlarged to accommodate 100 convalescent patients who were admitted from many parts of the country. Queen Victoria agreed to the name being changed to the Royal West of England Sanatorium and this was shortened to the Royal Hospital after the first world war. This hospital also closed when the new hospital opened in 1986.

### INTRAOPERATIVE SALVAGE AUTOTRANSFUSION

**J. Thompson**
Bristol

Intraoperative Salvage Autotransfusion (ATS) has been used in both emergency and elective surgery since 1886. Technical developments have been stimulated in the past by demands on available resources such as wartime. Latterly, concern over disease transmission and the immunological implications of transfusion has rekindled interest. The UK appears to be 3–4 years behind the USA in implementing ATS programmes and this reflects the spread of HIV.

The current state of the art involves three broad categories:

1. Simple reservoir systems (e.g. Solcotrans), where salvaged blood is reinfused directly, without washing, via a 40um filter. Systemic heparinisation is required for efficient use.
2. Cell saver devices (e.g. Haemonetics, Dideco). These devices inject heparin at the sucker tip and can be used in non-heparinised cases. A new, inexpensive, portable device has made this option more widely available.
3. Postoperative wound drainage collection. The Sorensen system is used to salvage difibrinogentated chest or mediastinal drainage. Recently an orthopaedic system has been introduced, and cell savers can be used for postoperative drainage.

The relative advantages, disadvantages, revenue implications and applications of these devices was presented.

### THE LIVES AND LEGENDS OF THE PATRON SAINTS OF SURGERY

**Mark W. Kissin**
Bristol

Although there are >4,500 Saints listed in the 'Roman Martyrology', for many all that is known is their era, place and mode of death and feast day. Throughout the Christian era, Saints have been adopted as Patrons by countries, trades, and as protection against disease. Patronage is usually related to the origin of the Saint, their most spectacular miracle or mode of death.

With the aid of the nuns of St. Vincent's, 265 Patron Saints of differing trades, 85 of countries and 67 related to medicine were identified. Of the latter group, 39 were Patrons of specific disease (e.g. St. Vitus and Chorea), and 28 of medical professionals (e.g. St. Catherine and the nursing service).

Twelve Saints were identified as Patrons of surgeons or surgical conditions. The most notorious being St. Agatha, Patron of breast disease, who was martyred by mastectomy.

The lives, legends and cults of the Patron Saints of General Surgery will be presented.

| SURGICAL SPECIALITY | DISEASE | PATRON SAINT |
|---|---|---|
| All surgeons | | Cosmas & Damian, Luke, Pantaleon |
| General Surgery | Breast disease | Agatha |
| | Cancer | Peregrine |
| | G-I Surgery | Erasmus |
| | Hernia | Osmund |
| | Haemorrhoids | Fiacre |
| ENT Surgery | Throat disease | Blaise |
| Eye Surgery | Eye disease | Lucy |
| Dental Surgery | Toothache | Apollonia |

